# The complete chloroplast genome of *Clematis hexapetala* (Ranunculaceae) and its phylogenetic analysis

**DOI:** 10.1080/23802359.2022.2079101

**Published:** 2022-06-02

**Authors:** Yi Cui, Yingxin Sun, Yanzhe Ding, Jing Liu, Zhongming Han, Yunhe Wang, Mei Han, Limin Yang

**Affiliations:** a College of Chinese Medicinal Materials, Jilin Agricultural University, Changchun, China; b State Key Laboratory of JLP-MOST for Ecological Restoration and Ecosystem Management, Changchun, China

**Keywords:** *Clematis hexapetala*, chloroplast genome, phylogenetic analysis

## Abstract

*Clematis hexapetala* Pall. (1776) is a traditional Chinese medicine belonging to the Ranunculaceae. In this study, the complete chloroplast genome was sequenced through Illumina platform, cp was circular DNA molecule of 159,538 bp in length with a typical quadripartite structure, consisting of four regions: two copies of inverted repeat region (IRs: 31,039 bp), a large single-copy (LSC: 79,333 bp) region, a small single-copy (SSC: 18,127 bp) region. The chloroplast genome encodes a total of 135 genes, including 91 CDS genes, 36 tRNA genes, 8 rRNA genes. Phylogenetic analysis based on complete genes shows that *C. hexapetala* closely related to *C. taeguensis* in the genus *Clematis*. This study improves our comprehension of the chloroplast genome and its phylogenetic relationships within Ranunculaceae.

*Clematis* L. is one of the largest genera in Ranunculaceae with about 280–350 species (Tamura 1987, 1995; Wang and Li 2005; Chen et al. 2021) most of which are woody or herbaceous vines, but a few are shrubs, subshrubs, or erect perennial herbs (Ghimire et al. 2020). Among them, *Clematis hexapetala* Pall. (1776) is distributed in the western of Jilin province, China, as well as in Korea, Mongolia, and Russia (Wang and Li 2005). *Clematis hexapetala* have been used for many years in China as an important traditional Chinese herbal medicine and is officially listed in the pharmacopeia of the People's Republic of China (National Pharmacopoeia Committee 2020). Pharmacological studies have shown that the dried roots and rhizomes of *C. hexapetala* has been shown to have activities against various human diseases and conditions, including analgesic, diuretic and anti-inflammatory agents and are known as “Weilingxian” (Dong et al. 2007; National Pharmacopoeia Committee 2020). At present, the research of *C. hexapetala* is mainly focused on physiological characteristics, chemical constituents and pharmacology (Dong et al. 2007; Cai et al. 2020; Zhang et al. 2021). However, there are few reports about classification and evolution of *C. hexapetala.* In this study, we assembled the complete chloroplast genome sequence of *C. hexapetala* based on Illumina platform sequencing and analyzed the characters of the complete chloroplast genome sequence and phylogenetic relationship of *C. hexapetala* to confirm its phylogenetic position and evolutionary relationship between the *C. hexapetala* and other Ranunculaceae species, which is valuable for future studies on molecular markers to be helpful for its evolution, genetics research and furtherly contributes to the classification.

Genomic DNA was extracted from the fresh leaves, which were collected from Medicinal Herb Garden of Jilin Agricultural University (JLAU; Accession number: *Y. Cui 2021008; Zeliang Lü, email:*lvzeliang@foxmail.com), Changchun, Jilin Provence, China (43°48′23″N, 125°24′57″E). The voucher specimen was preserved at the Herbarium of JLAU. The total genomic DNA was extracted with the QIA quick Gel Extraction kit (Qiagen, GER). A chloroplast genomic library was constructed with PCR technology and sequenced with Illumina Hiseq 2500 (Illumina, USA). We assembled these high Quality reads into complete chloroplast genomes using metaSPAdes9 (Nurk et al. 2017), furtherly judged whether the ring was formed, corrected the direction and judged the starting base position. Finally, 16,418,120 raw reads were obtained. The assembled chloroplast genome was annotated using the online annotation tool CPGAVAS2 (Shi et al. 2019). The complete chloroplast genome sequence with gene annotations were submitted to NCBI with the accession numbers OK217287.

The complete chloroplast sequence of the *C. hexapetala* was 159,538 bp in length and structure was a typical quadripartite, consisting of two inverted repeat (IRs) regions of 31,039 bp each, separated by a large single-copy (LSC) region of 79,333 bp, and a small single-copy (SSC) region of 18,127 bp. The overall GC content was 37.97%. The content distribution in each region of chloroplast genome is uneven. The GC content is the highest in IR regions (42.1%), the corresponding values of the LSC and SSC are 31.4% and 36.3%, respectively. The chloroplast genome contained 135 functional genes, including 91 CDS genes, 36 tRNA genes, 8 rRNA genes.

The phylogenetic analysis was generated based on the complete cp genome of *C. hexapetala* and other 14 species. Fourteen complete chloroplast genome sequences were downloaded from NCBI to further investigate the phylogenetic position of *C. hexapetala*. (*Oxygraphis glacialis* species were used as outgroups). The phylogenetic tree showed that *C. hexapetala* was closely related to *C. taeguensis* (Figure 1). The complete chloroplast genome sequence of *C. hexapetala* will provide a useful resource for the phylogenetic studies of Ranunculaceae.

**Figure 1.**
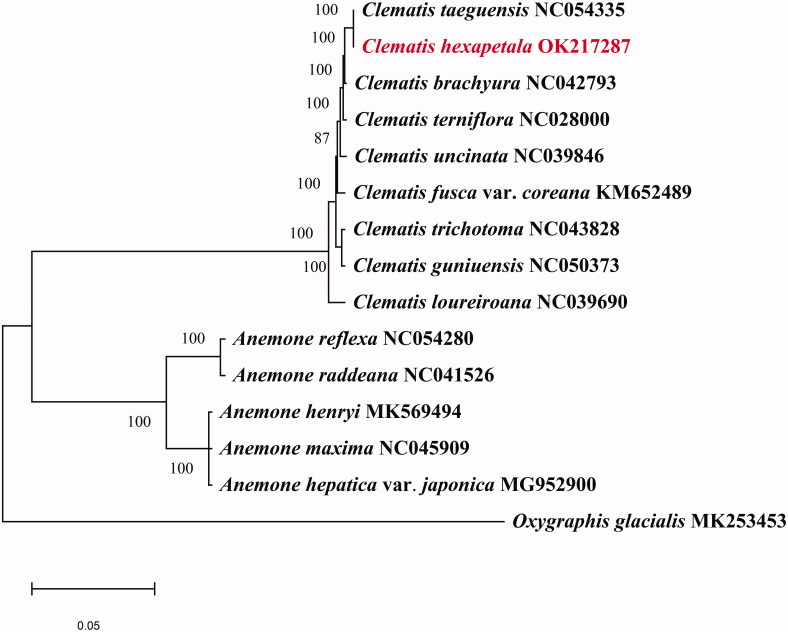
The phylogenetic tree was constructed using chloroplast genome sequences of 15 species based on the maximum-likelihood analysis using 500 bootstrap replicates. The numbers on the branches are bootstrap values.

## Ethical approval

This article does not contain any studies with human participants or animals performed by any of the authors. The specie in this paper is not endangered, protected, or personally owned.

## Authors’ contribution

Zhongming Han and Yunhe Wang conceptualized and designed research; Yi Cui analyzed data and wrote original draft of the manuscript; Yingxin Sun, Yanzhe Ding, Jing Liu, Yunhe Wang contributed to research materials and to the draft manuscript; Mei Han and Limin Yang reviewed and rewrote the manuscript. All authors read and approved the final manuscript.

## Data Availability

The genome sequence data that support the findings of this study are openly available in GenBank of NCBI (https://www.ncbi.nlm.nih.gov/) under the accession numbers OK217287. The associated BioProject, SRA, and Bio-Sample numbers are PRJNA764578, SRR15990422, and SAMN21529435, respectively.
